# Hospital Note

**Published:** 1901-12

**Authors:** 


### HOSPITAL NOTE.

The New York Orthopedic Dispensary and Hospital, No. 126 East 59th Street, New York, announces that the Surgeon-in-chief, Dr. Russell A. Hibbs, will give a course cf clinical lectures on orthopedic surgery at the institution, on Monday and Thursday afternoons, at five o'clock, from December 2 to January 2 (both inclusive). The course will be free to the medical profession and students.

